# Sustainable production of microalgal nanoparticles through green synthesis towards cancer treatment

**DOI:** 10.3389/fbioe.2025.1621876

**Published:** 2025-09-17

**Authors:** Vijay Kumar Garlapati, Swati Sharma, Deepak Sharma, S. P. Jeevan Kumar, Samuel Jacob, Arindam Kuila, Ashok Kumar Gupta, Abhishek Chaudhary

**Affiliations:** ^1^ Department of Biotechnology and Bioinformatics, Jaypee University of Information Technology, Waknaghat, Himachal Pradesh, India; ^2^ ICAR-Directorate of Floricultural Research, Pune, Maharashtra, India; ^3^ Department of Biotechnology, School of Bioengineering, College of Engineering and Technology, SRM Institute of Science and Technology, Kattankulathur, Tamil Nadu, India; ^4^ Department of Bioscience and Biotechnology, Banasthali Vidyapith, Tonk, Rajasthan, India; ^5^ Department of Civil Engineering, Jaypee University of Information Technology, Waknaghat, Himachal Pradesh, India

**Keywords:** green synthesis, microalgal nanoparicles, sustainability, cancer treatment, future prospective

## Abstract

Nanoparticle-based treatment is one of the rapidly growing research domains in cancer treatment due to its associated structural, targeted, and stability features. The conventional (chemical and physical) nanoparticle (NP) synthesis suffers from drawbacks such as toxicity, cost, and unsustainable process methodologies, which necessitate the urgent need for sustainable green approaches to nanoparticle synthesis for envisioned cancer treatment options. The green synthesis of microalgal NPs is a promising approach for obtaining NPs for cancer treatment. As a result, this review presents the synthesis mechanism of microalgal NPs and the factors affecting their green synthesis. The mechanism of action of microalgal NPs in cancer treatment has been discussed in relation to their cytotoxic effects. The sustainability features, probable quality control regime of green-synthesized microlgal NPs, along with the prospects of incorporating synthetic biology and utilizing genetically engineered microalgae, have been highlighted in the context of cancer treatment.

## 1 Introduction

Cancer is a vast concept that refers to a variety of disorders in which the body’s cells grow in an unrestrained manner. There are approximately 200 distinct forms of cancer, and specific cancerous cells can spread to other tissues, creating deadly metastases. Cancer is the most significant cause of mortality worldwide due to population expansion and aging. Because of this substantial degree of impact, cancer treatment has received much interest from the research community (Cooper et al., 2023). According to a report by the UN Health Department, cancer is the leading cause of death worldwide, contributing to nearly 10 million deaths in 2020 or nearly one in six deaths (Siegel et al., 2023). Due to the side effects of existing cancer treatments, it remains challenging to identify more effective therapies, and the development of novel anticancer drugs for anticancer therapy is critical for sustained advancement. It is well known that approximately 60% of hematology and oncology medications come from naturally driven sources, and 33.3% of the most often prescribed drugs are natural substances or derivative products. Because natural solid substances (e.g., polyketides, steroids, phenolic compounds, terpenoids, and antioxidants) have previously been identified in marine species, there has been enormous growth in the research on marine bioactive metabolites (Liu and Qin, 2023).

The method of monitoring and diagnosing diseases by applying nanotech for control is termed “nanomedicine” (Ma and Shi, 2021). Cancer medications that are pharmacologically active reach the tumor tissue with low specificity and dose-limiting toxicity during treatment. Intravenous (IV) and oral routes are two common drug delivery modalities. These approaches have several drawbacks. For example, oral administration of capsules and tablets may result in chaotic pharmacokinetics due to drug exposure to the body’s metabolic pathways. This can lead to the administration of higher-than-necessary doses, which can lead to increased toxicity. Traditional IV approaches are frequently even more difficult. Some traditional intravenous medicines have limited specificity, causing injury to healthy tissues. Nanoparticle-based conjugates are one of the best ways to deliver drugs to target tissues (Kandula et al., 2023).

**FIGURE 1:**
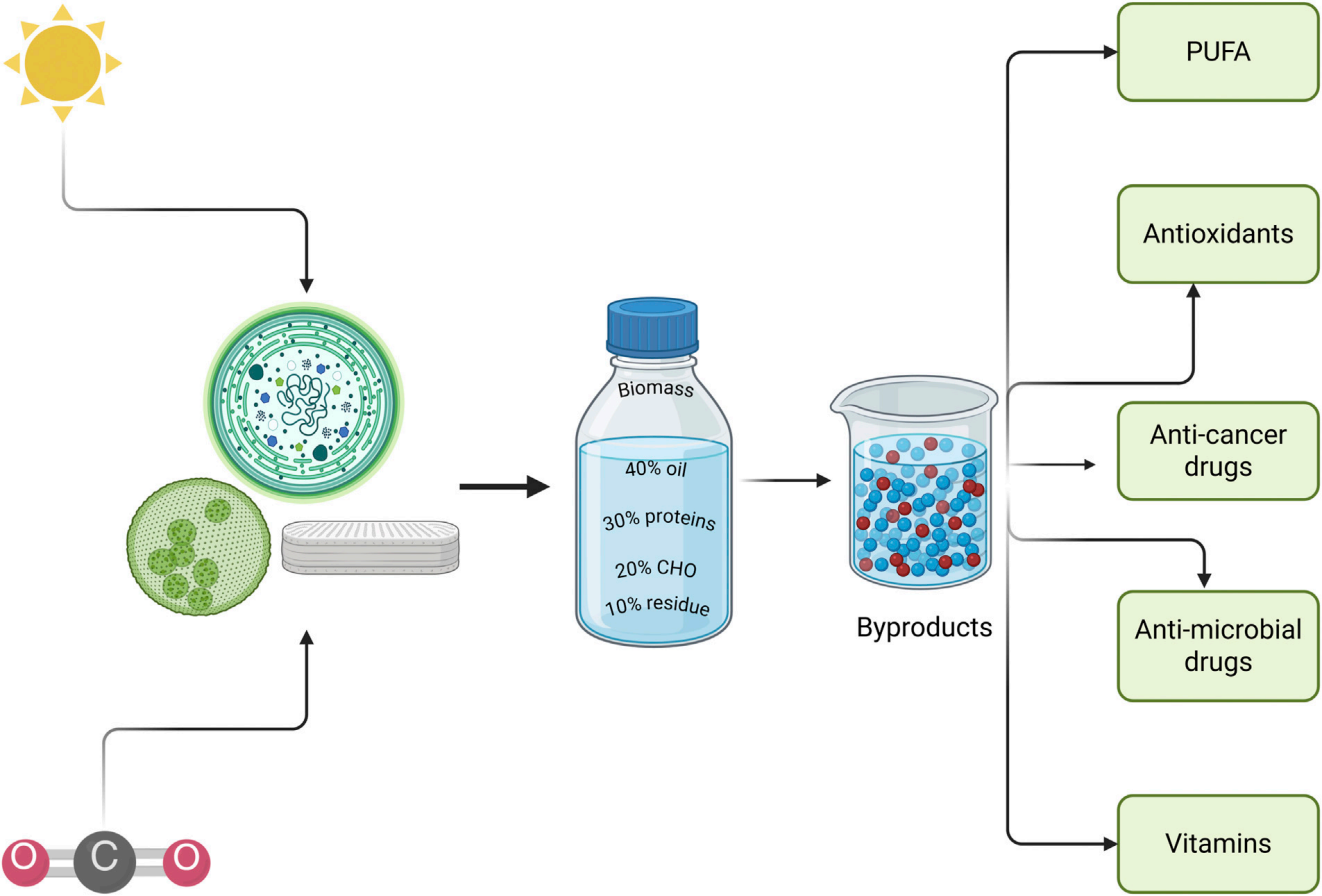
Microalgae as a source of different bioactive compounds (Created in https://BioRender.com).

The photosynthetic microorganisms of marine species, specifically microalgae, can be categorized as eukaryotic (green algae, diatoms) or prokaryotes (blue‒green algae), which are able to produce some of the important compounds of medical interest (Figure 1). Seven marine-derived drugs are available on the marketplace; out of the seven available drugs, four are anticancer drugs. There are nearly 26 marine natural compounds in medical testing, 23 of which exhibit anticancer properties. There are ongoing clinical trials of anticancer drugs produced by green algae (Al-Zahrani et al., 2021). Microalgae produce secondary metabolites, lipid derivatives, carbohydrates, and proteins with various structures. These compounds have been clinically tested, and the results indicate that these conventional treatments strengthen the immune system and assist in cancer elimination (Saadaoui et al., 2020). Halogenated compounds, fatty acids, peptides, steroids, carotenoids, etc., are produced by green algae; these molecules bind at various sites, suppress the mitotic cycle, and cause apoptosis due to cellular pathway activation. In addition to their anticancer effects, these compounds have antioxidant, antimicrobial and anti-inflammatory effects (Ruzik, 2023).

There has been an increase in research in the context of microalgal technologybecause of their rich nutritional bioactive componentsand most importantly, they are renewable, have a high growth rate, require no land, are easily cultivated and harvested, and can grow in all seasons. Some marine species are known to be beneficial health supplements. Antioxidants containing algae are indispensable in cancer therapies. Drugs used in chemotherapy eliminate cancer cells but stimulate the formation of highly reactive partially oxidized compounds called reactive oxygen species (ROS), which have harmful effects. Therefore, antioxidants are given alone or in combination with chemotherapeutics (Ferdous and Yusof, 2021). These antioxidants activate the defense system, prevent genomic instability caused by ROS, and inhibit the proliferation of cells (Abd El-Hack et al., 2019).

The bioactive compounds from microalgae include carotenoids, phycobilin, polyunsaturated fatty acids, polysaccharides, sterols, vitamins, enzymes, and proteins, which have multiple applications in the pharma and cosmetic sectors (Silva et al., 2022). Microalgae produce antioxidant compounds, e.g., microalgal-derived tetraterpenoids, which are antioxidantsthat exhibit antitumor activity (Ferdous et al., 2021). β-Carotene from *D. salina* has been reported *to have*immunomodulatory and anti-inflammatory effects and is atherapeutic agent for colon, prostate, breast, and lung cancers (Lee et al., 2020). In the cosmetic sector, algal carotenoids are antioxidants, anti-aging agents, and natural pigments (Foo et al., 2021). Algal bioactive compounds, such as α-tocopherol, ascorbic acid, and β-carotene, have shown promising effects on prostate cancer (PC-3) cells through their ability to reduce cell viability and increase reactive oxygen species (ROS) levels and lipid peroxidation (LOP) (Ayna, 2020). Another microalgal bioactive compound, namely, astaxanthin, has shown better antioxidant properties than carotenoids and helps to avoid cell and tissue damage; it is a therapeutic candidate for different malignant cells and has also been reported to act as an anti-aging agent through enhanced aerobic metabolism by preventing protein oxidative decomposition (Faraone et al., 2020; Zhuang et al., 2022).

Microalgae are a potent alternative source for sustainable nanoparticle (NP) synthesis. Low metal concentrations are necessary for microalgal cellular functions such as photosynthetic electron transfer, N_2_ assimilation, and cofactors for enzymatic reactions, with the foreseen synthesis parameters affecting their morphology and functions (Hamida et al., 2022; Hamida et al., 2020a; Jacob et al., 2021). Microalgal cells secrete metal chelating agents to mitigate high metal concentration toxicity, which aids in converting metals to nanosized metal nuclei, which is the basis for NP synthesis (Hamida et al., 2020b; Bin-Meferij and Hamida, 2019). Algal metallic NPs of 1–100 nm in size, such as silver (Ag), gold (Au), and platinum (Pt), have been reported to have significant therapeutic efficacy in treating different health ailments, such as cancers (Abed et al., 2022; Xue et al., 2021)), infectious diseases (Lin et al., 2021; Baby and Reji, 2022), and diabetes (Koushki et al., 2021). Microalgal NPs serve as potent candidates in cancer treatment due to their physicochemical properties (small size, large surface area, and surface chemistry), which aid in quickly penetrating cell membranes and organelles toward programmed cell death (Ferdous and Nemmar, 2020). AgNPs have been reported to have promising effects on cancer cells (Nguyen et al., 2021; Haque et al., 2021), with high therapeutic efficacy against MCF-7, Caco-2, HepG2, and HCT-116 malignant cells (Hamida et al., 2020c; Acharya et al., 2022; El-Naggar et al., 2018; Rana and Prajapati, 2023; Sharma et al., 2023; Sharma et al., 2022).

The present review focused on the green synthesis of microalgae-based nanoparticles towards cancer treatment by emphasizing microalgae as a potential for.

## 2 Green synthesis of microalgae-based NPs for cancer treatment

NPs are beneficial because they have a large surface area due to their small size (in Nanos), and they can easily cross the cell-tissue barrier to reach their target site. Some natural anticancer drugs, such as camptothecin, may not be used due to their poor solubility, and nanotechnology offers novel solutions to address such difficulties (İnan et al., 2021; Sun et al., 2021). Hydrophobic drugs are encapsulated in nanoparticles, making them soluble; on administration, such drugs are released from the nanoparticle onto the target. Nanoformulations carrying anticancer compounds offer certain advantages, such as better solubility, proper drug accumulation at the target site, enhanced half-life, less toxicity and low cost and stable release of drugs (Heinemann et al., 2021). NPs help bioactive compounds escape the immune system and reach their target to eliminate diseased cells by bypassing biological barriers (Khan et al., 2019).

**FIGURE 2:**
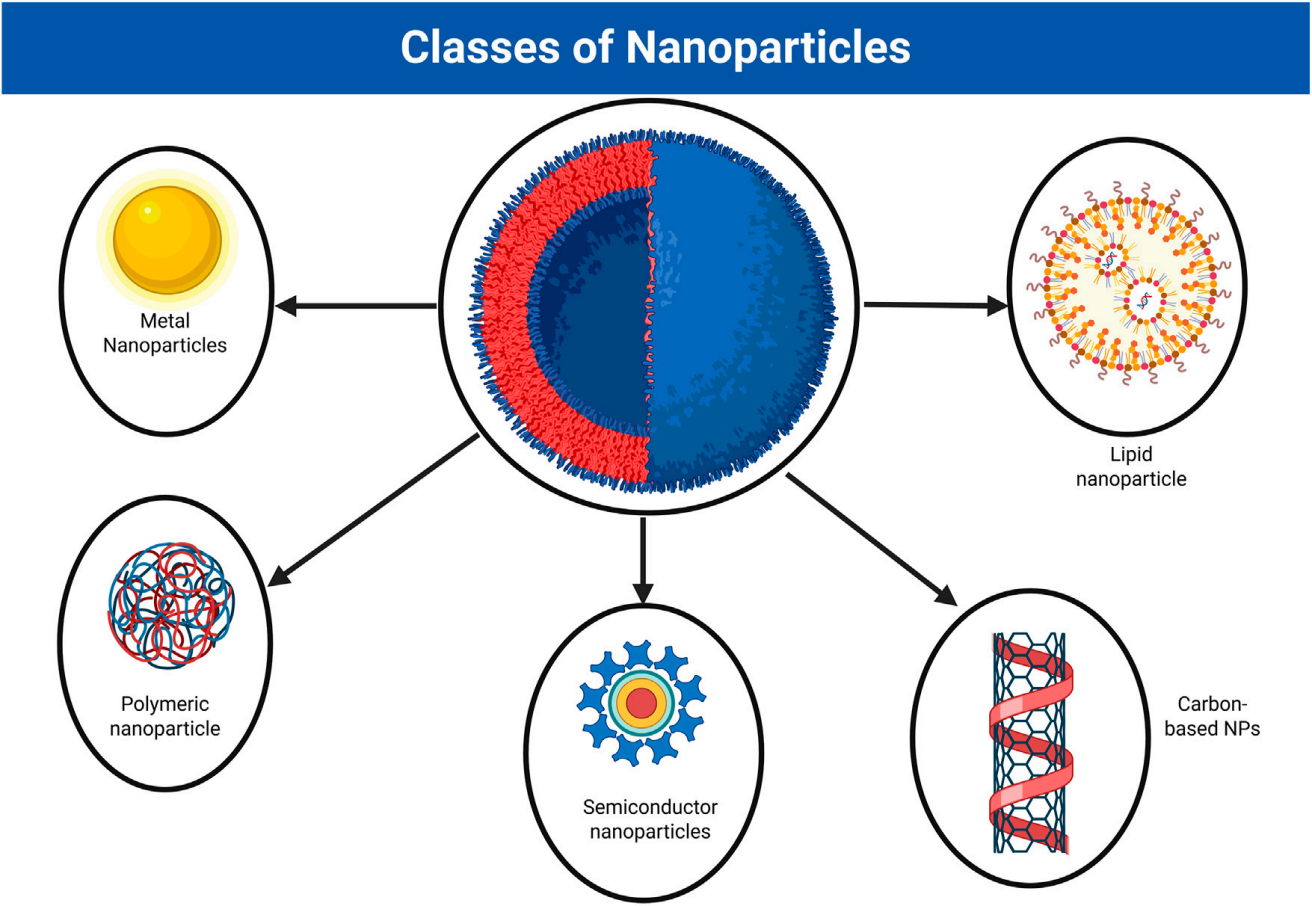
Different classes of nanoparticles (Created in https://BioRender.com).

Several classes of nanoparticles are known, such as (a) carbon-based nanoparticles, which have applications in biomedical fields and biosensors; (b) metal-based NPs, which are used in drug and gene delivery, radiotherapy, and anticancer; (c) polymeric NPs, which are used in biosensors and have environmental and agricultural applications; (d) ceramic NPs, which are used in bone repair; (d) lipid-based NPs, which can overcome biological barriers in the case of cell transfection; and (f) semiconductor NPs, which may have diodes, solar cells, and laser technology applications. The different classes of nanoparticles are depicted in Figure 2.

Biosynthesized nanoparticles such as microalgae-based green synthesized NPs are preferred for cancer treatment becauseof their eco-friendly synthesis without the requirement of high pressures, high temperatures, and toxic chemicals (Khan et al., 2019; Alsammarraie et al., 2018). The microalgae processing for green nanoparticle synthesis consists of microalgal cell cultivation (upstream processing, USP) under stress conditions (Kaushik et al., 2023) and harvesting of microalgal biomass (downstream processing, DSP) (Katiyar and Arora, 2020; Khoo et al., 2020; Tang et al., 2020; Khanra et al., 2018; MatAron et al., 2021). The microalgae-based green synthesized gold nanoparticles (AuNPs) and silver nanoparticles (AgNPs) found to be effective as anticancer agentsthose findings were tabulated in Table1.

**TABLE 1:** Microalgal-based nanoparticles and their role in cancer therapy.

Microalgae	NPs	Applications	References
*Dunaliella salina*	Gold	Anticancer activity against MCF7 cell line	Singh et al. (2019)
*Trichodesmium erythraeum*	Silver	Active against cervical and breast cancer	Sathishkumar et al. (2019)
*Dictyosphaerium sp.DHM1* *Dictyosphaerium sp.DHM2* *Dictyosphaerium* sp. *DHM3*	Silver	Active against breast cancer (MCF7) and hepatocellular carcinoma (HepG2)	Khalid et al. (2017)
*Ulva rigida* *Cystoseira myrica* *Gracilaria*	Silver	Active against MCF-7 (breast cancer)	Algotiml et al. (2022)
*Chaetomorpha ligustica*	Silver	Active against colon cancer cell line HT29 and HCT116	Al-Zahrani et al. (2021)

Various microalgal species, such as *Chlorella, Spirulina, and Scendesmus,* were utilized for green synthesis of AgNPs. The process involves the suspending of microalgal biomass in an aqueous silver nitrate solution (1 mM). The synthesized AgNPs were characterized by transmission electron microscopy (TEM), UV spectroscopy, energy dispersive X-ray energy dispersive spectroscopy (EDX) followed by the evaluating the cytotoxicity of the synthesized NPs for anti-cancerous activity (Tran et al., 2023). The green synthesized gold nanoparticles (AuNPs) of *D. salina*, includes the steps of cultivating the microalgae in MJM media followed by harvesting of microalgal biomass, suspending the aqueous microalgal biomass (20%) in aqueous silver chloride (1 M), and finally centrifuged to get the AuNPs. The green synthesized AuNPs were further characterized by TEM, FTIR, and XPS (Singh et al., 2019).

AgNPs may act against virus-, cancer-, bacteria-, and fungus-infected cells. Muneebaetal (İnan et al., 2021), used the DHM1, DHM3, and DHM3 strains for microalgalNP synthesis to assess their activity against a cancer cell line (MCF7). As a part of the procedure, silver nitrate solution (5mM, aqueous) was added to the microalgae-derived ethanolic extract (5:1). On overnight incubation, a color change was observed from green to yellow and AgNPs were obtained by centrifugation. Sathishkumar et al. (2019) prepared AgNPs from aqueous extract using *Trichodesmium erythraeum* via an environmentally friendly method that was found to have anti-proliferative and antioxidant properties. Algotiml et al. (2022) biosynthesized nanoparticles from *Ulva rigida, Cystoseira myrica, and Gracilaria foliifera* and tested them for antimicrobial and anticancer activity. The major algal extract components included fatty acids, amide proteins, terpenoids, flavonoids, polyphenols, fluoro aliphatic compounds, pyruvic acid, volatile compounds, and alkalines. AgNPs exhibited antimicrobial and antifungal activity against foodborne microbes and pathogenic fungi, respectively. These AgNPs were proven to be antidermophytes in the case of skin infections and anticancerous against breast cancer (MCF7) cell lines. Al-Zahrani et al. (2021) synthesized AgNPs by using the microalgae *Chaetomorpha ligustica*. These compounds were effective against HCT116 and HT29 (colon cancer cell lines) cells. *Chaetomoroha ligustica* extract and its nanoparticles proved to be cytotoxic, but the cytotoxicity depended on the dose. Several other biosynthesized nanoparticles prepared from *Bifurcaria bifurcate*, *Chlorococcum humicola, Galaxuara elongata*, *Sargassum plagiophyllum*, *Amphora-46*, *Caulerpa racemose, Microcoleus sp., and Ulva fasciata* are known for their antibacterial activity (Al-Radadi et al., 2022; Zaman et al., 2020).

### 2.1 Factors affecting the green synthesis of microalgae-based NPs

The optimal yield of microalgae-based NPs depends on the synthesis factors, namely, temperature, pH, reactant concentration, reaction time, capping agent, and choice of organism. These factors may affect the shape, size, and stability of nanoparticles, determining nanoparticle toxicity.

#### 2.1.1 Temperature

Chemical methods such as electrochemical and solvothermal methods are highly influenced by temperature. Physical processes require a temperature of 350 °C, whereas chemical methods require a lower temperature. A temperature of 100 °C is needed for the synthesis of microalgal NPs. At high temperatures may increase the reduction rate (Shanmuganathan et al., 2023).

#### 2.1.2 pH

pH affects the shape and size of NPs; a low pH causes the SPR peak to widen and shift toward a longer wavelength region, producing a variety of NPs (often triangular or circular, for example). In contrast, a high pH is ideal for making small NPs and promotes the formation of spherical NPs. NPs show additional stabilization in alkaline or acidic environments. Large pearl-sized NPs were produced in abundance under alkaline conditions and were far more stable than the clustered NPs made under acidic conditions (Zhang et al., 2022).

#### 2.1.3 Reactant concentration

Varying effects on the generation of NPs can be caused by different reactant concentrations in an algal extract. The impact of reducing agent concentration on the size and quantity of selenium particles produced by *Chlorella vulgaris* extract was investigated. Transmission electron microscopy (TEM) analysis of the time-dependent creation of nanoparticles revealed a critical role for multiple twinned particles (MTPs) in this process. Additionally, it was discovered that the development of single-crystalline selenium nanotriles was caused by the sluggish nature of the reaction and the influence of the shape and direction of the extract (Dinc et al., 2022).

#### 2.1.4 Reaction time

Reaction time is a critical factor in microalgal NPs synthesis. The same experiment can produce varied particle sizes if the reaction time is altered. The algal extract used to synthesize the NPs began to develop in 2 min and produced spherical NPs with a mean size of 12 nm after 5 min. Additionally, the progressive increase in contact duration and interaction between microalgae and silver ions (Ag^+^) at room temperature results in an increase in the SPR peak intensity and the rapid biosynthesis of non-agglomerated AgNPs (Moraes et al., 2021).

#### 2.1.5 Choice of the microalgal strain

Cost-effective NPs synthesis depends not only on chemical-physical parameters but also on the chosen microalgae based on critical intrinsic properties such as biochemical pathways, growth rate, and enzyme activities; (ii) the size of the inoculum; and (iii) the selection of biocatalysts, which is essential for accelerating the rate of reaction (i.e., reduction). Whole cells and enzymes can all be employed as biocatalysts. Live whole cells are desirable because these coenzymes are expensive and may be recycled along the route, demonstrating their enormous efficacy (Moraes et al., 2021).

#### 2.1.6 Capping agent

The stability of NPS should be improved to prevent aggregation and oxidation, particularly by utilizing organic protective ligands whose head group binds to metal NP surfaces with high affinity to stabilize highly reactive surface atoms. The alkyl spacer between the head and tail groups of the ligand is thought to act as a capping shell and regulate the interparticle spacing. Furthermore, the surface reactivity and solubility of NPs are greatly influenced by the functional tail groups of the ligand. Importantly, it was discovered that the kind of ligand (such as disulfide, ammonium, thiol, or citrate) and the level of ligand capping, along with the synthetic conditions used, could systematically change the size, shape, and ligand-to-metal ratio of the NPs and directly affect their chemical and physical (such as electronic and optical) properties (San and Shon, 2018).

## 3 Synthesis mechanism of microalgal NPs

**FIGURE 3:**
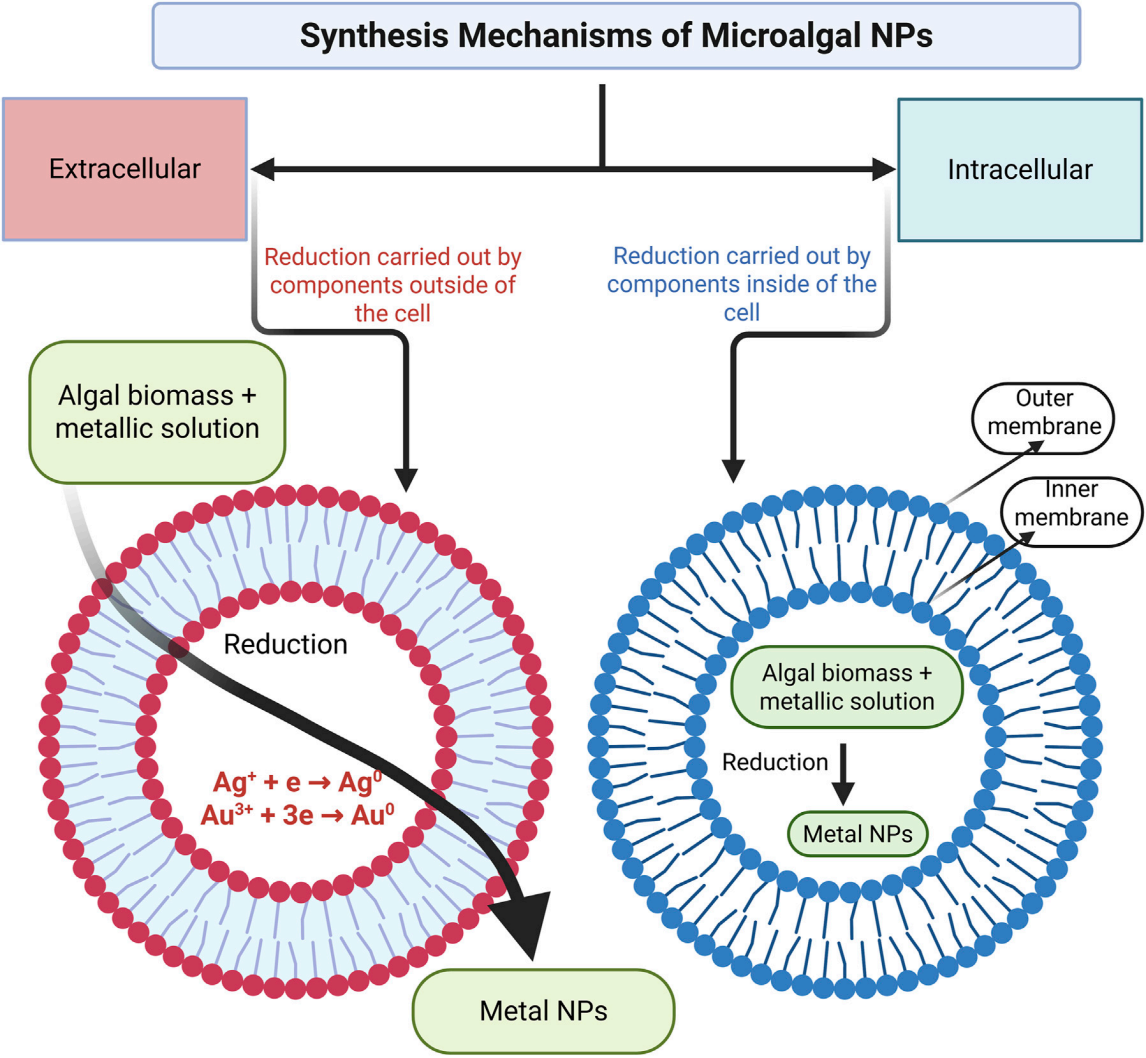
Extracellular and Intracellular synthesis mechanisms of microalgal NPs (Modified and adapted from (Chan et al., 2022)) (Created in https://BioRender.com).

Microalgae are used to prepare metallic nanoparticles that have applications in anticancer therapy. Phytochemicals in microalgae contain functional groups such as carboxyl, amino, and hydroxyl groups, which assist in reducing metals and serve as capping agents (providing coatings on nanoparticles). Nanoparticles of silver, cadmium, gold, lead, and silicon-germanium can be prepared using marine species (Restrepo and Villa, 2021). Green nanoparticle formation can be achieved via (a) intracellular synthesis, where compounds inside the cell carry out reduction; or by extracellular synthesis, where compounds outside the cell carry out reduction (Figure 3). In intracellular NPs synthesis, the metal ions taken by microalgal cells are reduced to NPs with the aid of microalgal metabolites, followed by NPs extraction from within the microalgal cells. In contrast, the extracellular NPs synthesis and stabilization proceed with the reduction of metal ions outside the microalgal cells (on the cell surface or algal extracts containing solutions) with the aid of secreted microalgal metabolites. In both approaches, microalgal metabolites/bioactive compounds act as reducing agents (by donating electrons, which reduces the metal ions to NPs synthesis) and capping agents (to stabilize and prevent clumping). The microalgal NPs synthesis also relies on the growth characteristics of microalgae and on the environmental factors (metal ion concentration, pH, temperature, etc.) which dictate the structural and stability features of synthesized NPs for tailored biomedical applications (Restrepo and Villa, 2021).

**FIGURE 4:**
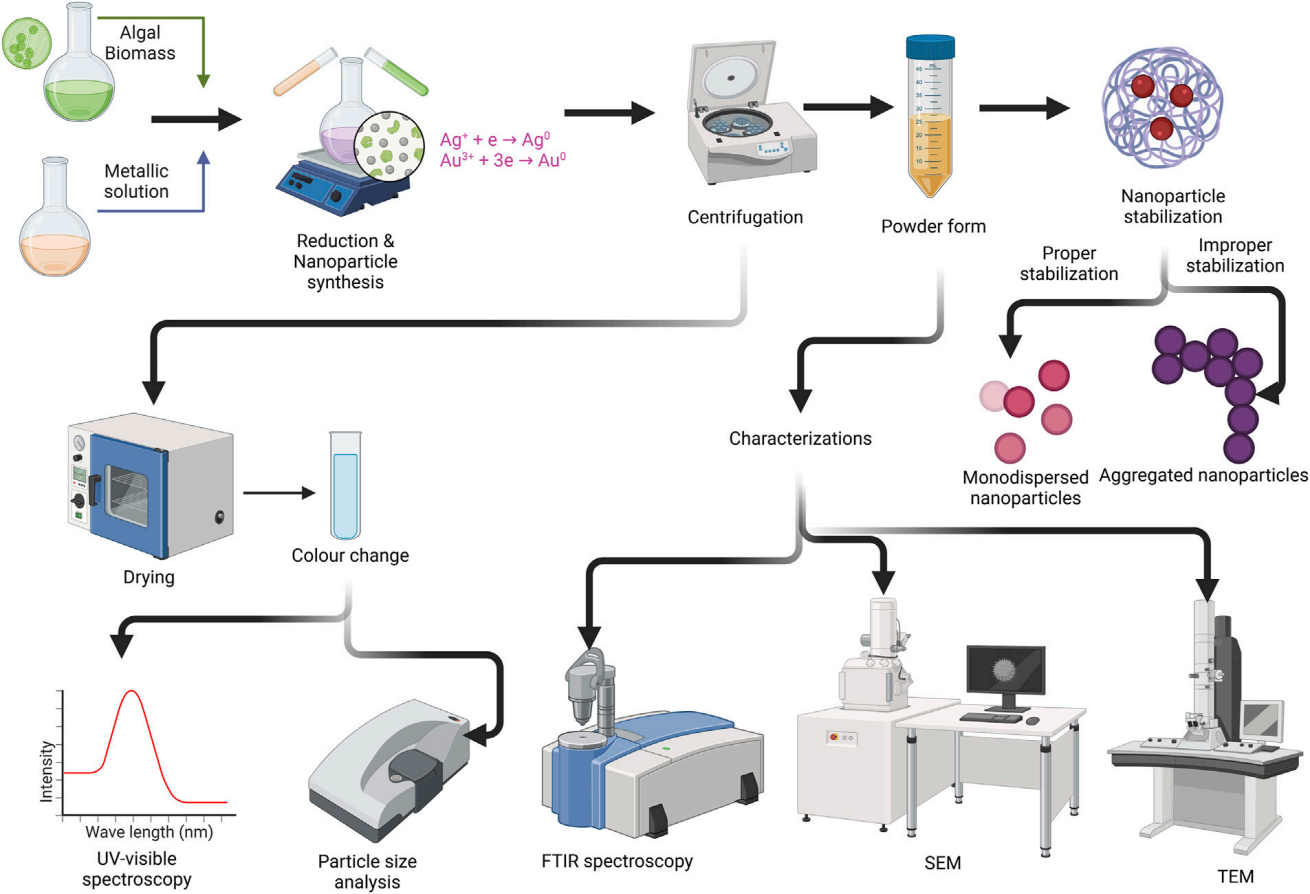
Schematic representation of microalgal NPs synthesis (Modified and adapted from (Chan et al., 2022)). (FTIR; Fourier Transform Infrared, SEM: Scanning Electron Microscopy, TEM: TransmissionElectroscopy Microscopy) (Created in https://BioRender.com).

The bioreduction process includes activation, growth, and termination. For instance, (a) during activation, metal ions are reduced, and reduced metal ions undergo nucleation; (b) during the growth phase, small nanoparticles aggregate into large particles marked with thermodynamic stability; and (c) termination is marked by biomineralization (inorganic (metal)-organic (microalgal assisted) composite) and nanoparticle stabilization (Figure 4). The formation of nanoparticles from *Tetraselmisko chinens*is and *Sargassum muticum* occurs via intracellular and extracellular synthesis (Chan et al., 2022). The general procedure for generating microalgal-based nanoparticles includes major steps, *viz.*, (1) the addition of a microalgal extract to a metal ionic solution where the reduction of the metal ion is caused by the functional nature of the microalgal compound; (2) the incubation of the sample after centrifugation, where successful reduction is marked by a change in the color of the solution (e.g., yellow to purple); (3) characterization, where transmission electron microscopy (TEM) reveals the size of the nanoparticles, energy dispersive analysis (EDX) confirms the presence of an element (e.g., silver), Fourier transform infrared (FTIR) spectroscopy reveals the type of biomolecule responsible for the stabilization of the AgNPs, and UV-VIS spectra determine the structure and properties of the nanoparticles (Singh et al., 2019).

Among different microalgae, the selection of microalgal species depends on the gamet of packed biomolecules posses by particular algal species, which dictates the metal ion stabilization towards NPs synthesis, and also on the type of expected metallic NPs with intended application domain. The selection of microalgae for NPs synthesis includes a systematic process of identifying the required metallic NPs for targeted application, with concrete proof of microalgal species for synthesizing particular metallic NPs. Once the type of NPs and microalgal species are identified, growth kinetics and particular metal uptake by selected microalgal species are determined, which follows the optimization of NPs synthesis reaction conditions (temperature, pH, and reaction time) towards better size, shape, and functionality. Finally, the synthesized microalgal NPs have to be characterized to determine the different structural and chemical properties for better suitability for targeted biomedical applications (Arteaga-Castrejón et al., 2024).

## 4 Characterization of the synthesized microalgal NPs

**FIGURE 5:**
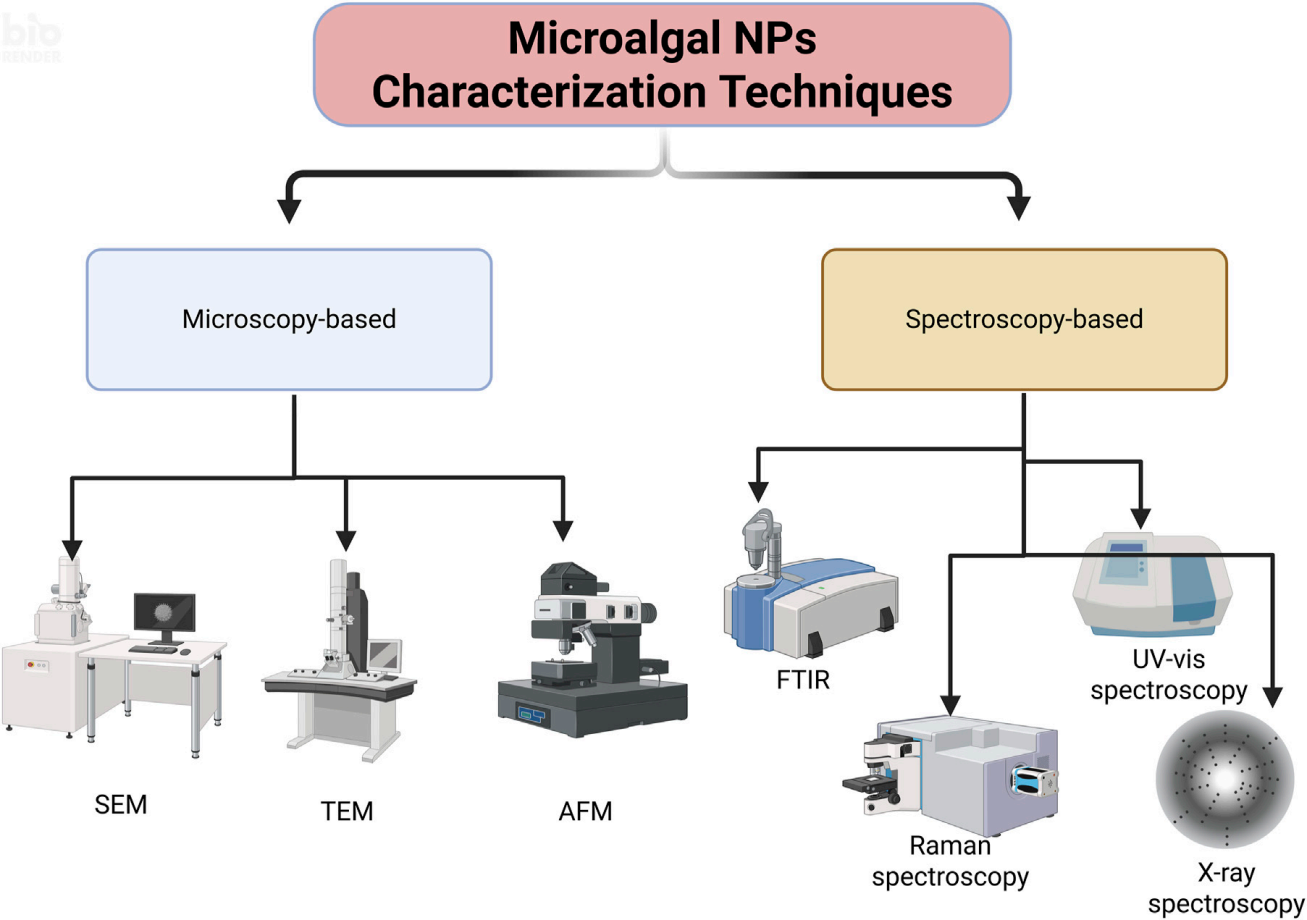
Microscopy- and spectroscopy-based characterization techniques for Microalgal NPs (SEM: Scanning Electron Microscopy, TEM: Transmission ElectroscopyMicroscopy,AFM:Atomic Force Microscope, FTIS: Fourier Transform Infrared Spectroscopy) (Created in https://BioRender.com).

Characterization of the NPs was carried out to determine the microscopic structure and material properties through microscopy-, spectroscopy- and X-ray-based techniques (Figure 5). Table 2 highlights the characterization techniques, principles, and use of methods in nanoparticle synthesis and sample preparation.

**TABLE 2:** Different characterization techniques of NPs and their features.

Techniques	Principle	Use in nanoparticle synthesis	Sample preparation	Advantages	Disadvantages	References
Transmission Electron Microscopy (TEM)	High energy electron beam penetrates the sample and electrons which are transmitted from the image are focussed using an objective lens	Determination of structure, size, shape and morphology of nanoparticle	Very thin samples are prepared such as by using ultramichrotome with knife made of diamond in cryogenic conditions	Imaging, diffraction and microanalytical information are easily produced and then combined to give detailed insights into the properties and behavior of NPs	Beam Damage that leads to permanent irreversible change in local chemistry and microstructure	Khalid et al. (2017)
Scanning Electron Microscopy (SEM)	Primary and secondary electrons produced on electron hitting the sample produce high resolution image	Determination of size and formation of high resolution image check, presence of ions along with EDX	A thin conducting material layer is provided to specimen, usually made of gold	Speedy imaging, quick results, time-efficient processing, and quick turnaround time	Coloured, non-conducting and samples with higher dimensions are difficult to analyze	Mourdikoudis et al. (2018), Sathishkumar et al. (2019)
Atomic Force Microscopy (AFM)	Surface sensing with an incredibly sharp tip on a micromachined silicon probe. This tip is used to raster scan across the surface line by line to picture a sample	Analysis of surface tension and surface roughness	The sample needs to prepare as a dry powder, evaporated suspension, bio-particle or carbon nanotubes	Imaging of nanoparticles of 0.5 nm–50^+^ nm and estimation of size distributions of NPs	During detection, the sample and tip can be damaged	Mourdikoudis et al. (2018)
Ultraviolet- Visible (UV-Vis) Spectroscopy	Works on the principle of interaction of light with matter. With increased light absorption, the energy content of the molecules/atoms will raise	Study of kinetic behaviour and indication of NPs specific wavelength	Organic and Inorganic samples need to be prepared in ethanol and water, respectively. UV detection uses quartz sample holders	Easy to use and quick analysis of NPs	The stray light produced by poor equipment design and other circumstances reduces the linearity range and absorbancy of the substance being measured	Khalid et al. (2017)
Raman Spectroscopy (RS)	Based on incident light dispersing in elastically as it interacts with vibrating molecules	Study of vibrational and rotational modes	Little to no sample preparation, directly can obtain the spectra from liquids, polymers, solids, papers, etc.	It can determine composition as well as structural arrangement of NPs	Needs highly optimized instrumentation, impurities in the sample could hinder Raman spectra	Mourdikoudis et al. (2018)
Fourier Transform Infrared (FTIR) Spectroscopy	Change in banding pattern of absorption indicates uniqueness in NPs composition	Identification of NPs nature, reducing metal ion and capping of reduced NPs	It makes use of powdered samples formed by mixing and grinding in KBr	It allows both qualitative and quantitative analysis, provides high signal to noise ratio	Difficult to analyze black materials and difficult to obtain spectrum for amorphous materials	Khalid et al. (2017)
X-Ray Diffraction (XRD)	Each element of the sample diffracts X-rays depending on type of atoms and atomic arrangement	Visualization of crystalline structure of NPs	It makes use of powdered samples formed by grinding using pestle and mortar. The sample holder is made of aluminum plate	Minimal sample is required for analysis	Too broad peaks are obtained for particle with size less than 3 nm	Khalid et al. (2017)
X-ray Photoelectron Spectroscopy (XPS)	The no. of electrons escaped from the surface of material and kinetic energy measurements gives the XPS spectra	Determination of surface functional state and elemental composition of NPs	Powdered samples are pressed into indium foil, power is dissolved in solvent and cast onto silicon wafer and sprinkled onto sticky carbon conductive tape for analysis	It doesn’t damage the samples and provide depth information	It requires solid dry form of samples and data interpretation is little difficult	Singh et al. (2019)

One of the advanced nanoparticle characterization techniques is nanoSIMS, which uses isotope labeling to analyze and study a complete biological system. It can distinguish between isotopes and combines fine spatial resolution with high atomic sensitivity. The zeta potential, also called the electrokinetic potential, is used to measure the electric charge on the nanoparticle surface. The zeta potential of the particles indicates nanoparticle stability (Fernandes et al., 2023). Other techniques include NMR, which is used to analyze NP size, atomic composition, electronic core structure, ligand density and ligand influence; dynamic light scattering (DLS), which is used to detect agglomerates and hydrodynamic size; SQUID-nanoSQUID, which is used to analyze magnetization remanence, blocking temperature and magnetization saturation; liquidTEM, which is used to study growth mechanisms, depict nanoparticle growth in realtime, superlattice formation and single particle motion; HRTEM, which is used to distinguish amorphous, poly- and monocrystallineNPs; electron diffraction, which is used to study lattice and long-range order parameters; cryo-TEM, which is used to explore aggregation pathways and complete growth mechanisms; electronomography, for 3D particle visualization, video, snapshots, and quantitative information at the atomic scale; electron backscatter diffraction (EBSD), which is used to examine microstructure, texture and defects in NPs; magnetic force microscopy (MFM), which provides information about the magnetic moment of NPs and differentiates them from nonmagnetic NPs (Mourdikoudis et al., 2018).

## 5 Microalgal NPs in cancer treatment

Microalgae are reservoirs of bioactive compounds such as sterols, polysaccharides, fatty acids, phycobiliproteins, phenolics, and vitamins that play major roles in preventing or curing cancer. Table 3 summarizes the different bioactive molecules produced by microalgae and their therapeutic potential.

**TABLE 3:** Microalgae derived bioactive molecules and their use in cancer.

Bioactive molecule	Therapeutics	Microalgae	References
Polysaccharide	➢ Inhibition of lung cell cancer growth ➢ Antitumor property ➢ Apoptosis of human hepatic carcinoma ➢ NPs exhibit antioxidant property	*Nostoc sphaeroids* *Chlorococcum* sp.*, C. pyrenoidosa, Scenedesmus* sp. *Tribonema* sp. *Navicula*	Li et al. (2018) (Sun et al., 2021) Zhang et al. (2019) Chen et al. (2019) (Fimbres-Olivarria et al., 2018)
Fatty acids	➢ Omega 3 fatty acid and omega ➢ Polyunsaturated fatty acid improve survival of breast cancer patients ➢ Reduction of biomarker and tumor marker in colorectal cancer	*α- linolenic acid- T. suecia* *H. pluvalis* *DHA- Cryptomonas* sp.	Darwito et al. (2019) (Song et al., 2019)
Sterols	➢ β- sitosterol reduce tumor growth in liver, lung, breast, colon, prostate cancer cells ➢ Sterol help in tumor growth inhibition by apoptosis	*N. salina* *S. maxima*	Elkhateeb et al. (2020) (Jiang et al., 2019)
Peptides and phycobiliproteins	➢ Anticancerous against laryngeal cancer cells and human melanoma ➢ PC downregulates metastasis and angiogenesis genes, it also possess efficacy of drugs involved in chemotherapy like betaine	*Porphyrayezoensis*	Zhang et al. (2020) (Jiang et al., 2019)
Carotenoids	➢ Lutein has apoptotic and antiproliferative property and downregulates genes (biomarker) involved in survival and growth of prostate cancer ➢ Anti-breast, anti-proliferative cancer activity ➢ Beta carotene kills human prostate cancer cells by apoptosis ➢ It suppresses stem colon cancer cells ➢ It also helps in suppression of lung cancer with vitamin A ➢ Astaxanthin downregulates anti apoptotic proteins and induces apoptosis and blocks metastasis angiogenesis in tissues ➢ Zeaxanthin is anticancerous, it is known to induce apoptosis in human melanoma cells ➢ Canthaxanthin causes apoptosis in melanoma cells and human colon adenocarcinoma ➢ Chemopreventive in oral cancer ➢ Fucoxanthin has antiproliferative property ➢ Antitumorous against lymphoma, leukemia, ostersarcoma, prostate, colorectal, breast, bladder, hepatocellular cancer ➢ FX exhibit anti cancer activity ➢ Violaxanthin (VLX) is antiproliferative ➢ Inhibit cancer and colon cancer cells ➢ Neoxanthin cause apoptosis in human prostate carcinoma cells, hinder tumor cells promotion stage ➢ Siphonaxanthin exhibit anti-angiogenic and anti-proliferative activity ➢ Apoptosis in human leukemia cells	*C. vulgaris* *C. sarociana* *C. protothecoids* *H. pluvialis* *N. salina* *Muriellopsis* sp., *Parachlorella* sp. *P. perpureum* *Aspergillus carbonarius* *P. tricornutum* *C. vulgaris* *C. protothecoids* *Codium fragile*	Chan et al. (2022) Chen et al. (2019) Heo et al. (2018) (Li et al., 2018) (Sanzo et al., 2018) Heo et al. (2018) (Cezare-Gomes et al., 2019) Faraone et al. (2020) Faraone et al. (2020) Kim et al. (2019) Faraone et al. (2020)
Vitamins	➢ Vitamin K and its derivatives are known to be anti cancerous against blood, colon, lung, liver, prostate, bladder. It activates apoptotic pathways in case of hepatocellular carcinoma ➢ Vit K2 cause death of breast cancer cells.It hinders apoptosis in prostate cancer cells ➢ Vitamin A decrease risk of lung, pancreatic, ovarian, cervical, gastric carcinoma risk ➢ It protects against digestive and hepatocellular carcinoma ➢ Vitamin C helps in preventing tumor metastasis and suppressing [progression of cancer by causing HIF1a degradation ➢ Ascorbic acid impede cancer growth ➢ It cause delay in colorectal, pancreatic, breast, melanoma tumors ➢ Vitamin D helps in increasing survival rate of patients with adenocarcinoma and digestive tract cancer and reduces mortality due to cancers ➢ Vitamin E reduces GI cancer and total cancer risk ➢ Tocotrienols suppress cancer cells ➢ Reduces risk of bladder cancer	*I.galbana* *P. lutheri* *T. obliqus* *S. costatum* *T. suecica* *I.galbana* *Dunaliella tertiolecta* *Chlorella sp.*, *Spirulina sp., I. galana, R. salina, T. suecia* *P.lutheri, T. suecia, I. galbana, S. coastum* *C. stigmatophora* *P. lutheri, I. galbana* *C. calcitrans, S. coastutum, T. suecia, D. tertiolecta*	(Ruiz-Domínguez et al., 2020) Xie et al. (2019) (Zhang et al., 2020) (Mustafi and Wang, 2020) Akiba et al. (2018) Keum et al. (2019) Urashima et al. (2020) (Akiba et al., 2018) (Keum et al., 2019) (Urashima et al., 2020) Urashima et al. (2020)
Coenzyme Q	➢ It increases nitric oxide and ROS and kills HCT116 (human colon cancer cells) ➢ Decreases inflammatory markers involved in hepatocellular carcinoma ➢ reduce adverse effects in breast cancer	*Porphyridium, purpeurem* *C. pyrenoidosa*	Jiang et al. (2019)
Minerals	➢ High mineral intake of zinc, manganese, magnesium, calcium, iodine with low intake of sodium, iron, phosphorus, and copper is known to minimize occurrence in postmenopausal women for colorectal cancer ➢ Daily selenium intake protects against cancer	*Macrominerals in N. granulate, T. chuii P. tricornutum* *Microminerals in P. aerugineum, Bortyococcusbraunii*	Swaminath et al. (2019)
Amino acids	➢ Glutamine and arginine intake is essential in patient undergoing chemotherapy to lessen inflammation	*C. vulgaris* *C. sarokiniana*	Lim et al. (2018)

### 5.1 Mechanism of action of microalgal NPs in cancer treatment

**FIGURE 6:**
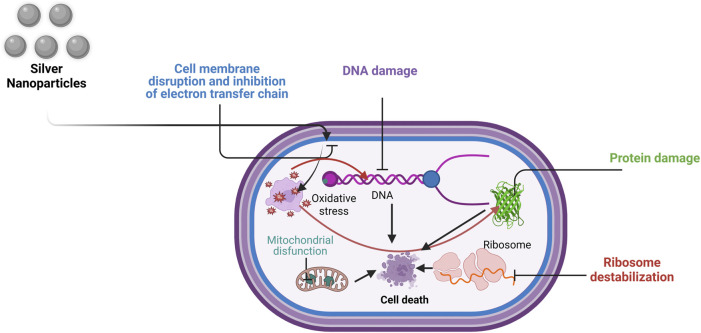
Mechanism of action of AgNPs in cancer treatment (Modified and adapted from (Hamida et al., 2022)) (Created in https://BioRender.com).

There are four major ways by which microalgae affect cancer cells. (1) Microalgae are known to decrease the binding capacity for tubulin polymerization, which inhibits the synthesis of microtubules (e.g., Cucarin A). (2) They alter the expression of COX-2, MMP-9, MMP-2, and ERK-2 (e.g., astaxanthin), which decreases invasion capacity. (3) They decrease VEGF, i.e., vascular endothelial growth factor (e.g., fucoidan), which results in antiangiogenic activity. (4) Increased fas, ICAM (intercellular adhesion molecule) and decreased bcl2 (e.g., C-phycocyanin), which cause the activation of caspase 2 3 4 6 8 9 10. Microalgae directly affect cancer in five ways. (1) PUFAs from microalgae, such as DHA, cause DNA fragmentation; (2) PUFAs, such as DHA, decrease the mitochondrial membrane potential. (3) PUFAs such as astaxanthin and DHA activate ERK, increasing p27. (4) Astaxanthin modulates NF-κB. (5) DHA increases cytochrome C, p53, and bax levels, leading to cell cycle arrest and an antiproliferative effect. All the above-mentioned factors lead to the apoptosis of cancer cells.The mechanism of action of microalgal AgNPs in cancer treatment is summarized in Figure 6 (Hamida et al., 2022).

### 5.2 Cell line studies and toxicity studies associated with the microagal NPs

The cell toxicity, genotoxicity, and immunotoxicity of NPs have been studied. As proven by many studies, these compounds are toxic to biological systems. The toxicity depends on the structure, size, and material from which the nanoparticle is made. To date, evidence has confirmed that biologically synthesized NPs, especially microalgal NPs, are less toxic to normal cells. Gold nanoparticles synthesized by using the microalga *D. salina* were tested for their effect on cancer (MCF 7) and normal (MCF 10A) cells. These compounds were shown to be cytotoxic to cancer cells and had no negative impact on the normal cell line (Singh et al., 2019). AgNPs made from the microalgae *T. erythraeum* caused antiproliferation in MCF-7 and HeLa cell lines, and they were not harmful to normal cells (Sathishkumar et al., 2019). AgNPs made using *U. rigida* were lethal to MCF-7 cells, a breast cancer cell line, and were not cytotoxic to normal cells. Because the core of gold NPs is inert and nontoxic, they are believed to be relatively harmless. On the other hand, metallic NPs are highly cytotoxic to normal cells; for example, aluminum oxide NPs decrease the viability of cells, increase oxidative stress, alter mitochondrial function, and change protein expression in the blood‒brain barrier (Algotiml et al., 2022). Copper NPs cause impairment in the kidney, spleen, and liver. Metallic AgNPs accumulate in many organs and can cross the BBB and reach the brain. After exposing rats to silver-based NPs by inhalation or subcutaneous injection, AgNPs have been found in several organs, including the lungs, spleen, kidney, liver, and brain. Furthermore, these NPs have shown greater ROS formation and cell survival toxicity. The most prevalent harmful effects of zinc-based nanomaterials, including cell membrane damage, cytotoxicity, and enhanced oxidative stress, have been described in diverse mammalian cell lines. Exposure of human mesothelioma cells and rodent fibroblasts to high concentrations of zinc oxide nanoparticles caused cell death (Hamida et al., 2022).

## 6 Technical challenges in green synthesis of microalgal NPs

The green synthesis of microalgal NPs process starts with the culture of microalgal cells and harvesting. Batch and Fed-batchor continuous cultivation modes are the most common techniques. Biomass is collected and processed during the cultivation phase. The cost of biomass harvesting is estimated to be nearly 30% of the price of microalgal downstream processes. As a result, the high cost of harvesting is one of the most significant bottlenecks in commercializing microalgal processes. Due to the density of microalgal cells during growth, the medium is often low, and most microalgal cells have a negative charge, which causes them to be suspended; moreover, the microalgal harvesting procedure is costly and energy-intensive (Jacob et al., 2021).As a part of harvesting, several harvesting procedures were reported such as sedimentation, ultrasound, centrifugation, filtration, and flotation, to maximize algal biomass yield. Nonetheless, these approaches are not as practical as flocculation because of their high cost and lower efficiency. The flocculation harvesting process is far more comfortable and efficient than previous methods, yet there are still many hurdles to overcome. On the other hand, utilizing flocculants to collect microalgal biomass can pollute slurry concentrates, lowering the market value of algal biomass and making it difficult to isolate valuable microalgal compounds. As a result, the only method to reduce harvesting costs is to improve harvesting technology (Halder and Azad, 2019).

The application of NPs in biomedical applications is the most vulnerable circumstance. The interaction between nanotoxicity and its biomolecules has been the subject of various studies. Nonetheless, assessing and validating nanotoxicity in a living system is complex. The assessment of toxicity and the challenges in identifying the influence on living systems are historic. Researchers face challenges when evaluating nanomaterials in solution-based, powder form and dealing with biological systems using various methodologies for toxicity evaluation. Evaluation tools and characterization procedures are critical for overcoming these hurdles, whereas cytotoxic assays consider nanoparticle shape, size, and morphology (Gupta et al., 2019).

## 7 Trends, scope and sustainability aspects of green synthesis of microalgal NPs

Since the discovery of nanoparticles, physical and chemical production methods have been prominent. In 2009, a wave led to both increases in nanoparticle synthesis and the adoption of biological synthesis methods. There has been an increase in the green synthesis of NPs owing to their sustainability and advantages. By emphasizing the green synthesis of NPs by microalgae, numerous microalgae have been explored for their ability to produce nanoparticles (Bao and Lan, 2019).

Despite their production, what remains unexplored is the application part of synthesized NPs. Research has yet to be carried out to determine their specific role in diagnostics, specifically in diagnosis of cancer. Table 4 summarizes the different clinical application status of green synthesized microalgal NPs. The production of reported microalgal NPs has increased the scope of further research that can focus on exploring the potential uses of these NPs in diagnostics and cancer therapy.

**TABLE 4:** Microalgal NP’s and its clinical application status (Bao and Lan, 2019; Patil and Kim, 2018).

Microalgae	NPs	Morphology Size (nm)	Application
*Chlorella vulgaris*	Gold	40–60	NA
*Chlorella vulgaris*	Palladium	2–15	Catalytic
*Chlorella vulgaris*	Gold	9–11	Antibiotic
*Chlorella vulgaris*	Silver	NA	NA
*C. vulgaris*	Silver	8–20	NA
*C. vulgaris*	Silver	50–70	NA
*C. pyrenoidosa*	Silver	2–15	Photocatalytic and antimicrobial
*Euglena gracilis*	Ferri-hydrite	0.6–1	NA
*E. gracilis*	Silver	6–24, 15–60	NA
*E. intermedia*	Silver	6–24, 15–60	NA
*Nanochloropsisoculata*	Manganese dioxide	NA	Lithium ion batteries
*Scenedesmus* sp.	Silver	15–20	Antibacterial
*Tetraselmissuecica*	Gold	79	NA
*Leptolyngbya tenuis*	Gold	NA	NA
*Diadesmis gallica*	Gold	9–27	Antibiotics
*C. chthonoplastes*	Gold	10–30	NA
*Nostoc ellipsasporum*	Gold	8–42	NA
*Eolimna minima*	Gold	NA	NA
*Euglena gracilis*	Gold	varied	NA
*Coelastrella* sp.	Gold	−30	Antioxidant
*Cosmariumimpressulum*	Gold	varied	NA
*Spirogira insignis*	Silver	30	NA
*Chlorococcum humicola*	Silver	2–16	Antibiotic
*Chlamydomonas* *Strain CC-124*	Silver	5–35	NA
*Microcoleus* sp.	Silver	44–79	Antibiotic
*Neochloris oleoabundans*	Silver	16.63	Antibiotic
*Chlorococcum humicola*	Silver	NA	Antibiotic
*Scenedesmus* sp.	Silver	5–10	Antibiotic
*Neochloris oleoabundans*	Silver	NA	NA
*Acutodesmus dimorphous*	Silver	5–20	Antioxidant
*Amphora - 46*	Silver	20–25	Antibiotic

### 7.1 Sustainability features associated with the green synthesis of microalgal NPs

Adopting green synthesis of microalgal NPs route brings economic and environmental sustainability features to the process. In the context of the environment, sustainability involves making efficient use of resources and keeping them for use by future generations. Microalgae grow with the aid of sunlight, carbon dioxide and inorganic nutrients such as nitrogen and phosphate. Utilization of atmospheric CO_2_ and inorganic nutrients of wastewater by microalgae makes the process more sustainable in nature (Chan et al., 2022). This sustainability feature of green synthesized microalgal NPs outperforms the existing chemical- and physical-based NPs synthesis techniques (sol-gel technique, nonsputtering, reduction, and electrochemical methods) by avoiding the usage of toxic, expensive reagents, high energy, pressure requirements of process, and associated intricacy of the separation process (Chan et al., 2022). The microalgal biomolecules serves as the capping agents for stabilization of synthesized NPs in case of green synthesis of microalgal NPs which aligns with green chemistry fundamentals (Mondal et al., 2023). The envisaged conditions of microalgal NPs green synthesis contribute to a lower environmental impact, which also aids in the cost-effectiveness of the process. Moreover, choosing microalgal route for NPs synthesis contributes towards the CO_2_ sequestration, coupled with wastewater treatment aspects. Being a biological process, the microalgal route of NPs synthesis results in minimal hazardous waste generation with bioremediation potential and a scalable process (by employing photobioreactor systems) along with the better economics of value-added byproducts side chains. These economic and environmental aspects make green synthesis of microalgal NPs, a viable alternative for conventional chemical and physical approaches for NPs synthesis, offering the advantages of sustainability and industrial feasibility (Mondal et al., 2023).

## 8 Quality control aspects of green synthesized microalgal NPs

NP use is divided into two primary categories: pharmaceutical and medical. The components and procedures utilized in drug production are subject to quality control. It is crucial to accurately assess formulation excipients and active pharmaceutical ingredients (APIs) for optimizing and evaluating preformulations. To guarantee the safety and efficacy of medications throughout the regulatory timeframe, they must be of sufficient strength, purity, quality, and potency. Many nanostructure systems, such as liposomes, nanoemulsions, dendrimers, nanocrystals, and metal oxides (zinc oxide, superparamagnetic iron oxide, titanium dioxide), have been approved by the FDA. The European Medical Agency and European Commission designated doxorubicin polyisohexylcyanoacrylate nanoparticles for treating hepatocellular carcinoma as orphan drugs and awarded this classification to the Bio Alliance in October 2004. The first nanodrug to receive FDA approval was Doxil in 1995. Dosage forms contain pegylated liposomes that carry the chemotherapeutic drug doxorubicin. An injectable amphotericin liposome is known as an AmBisome^®^ (Taghizadeh et al., 2021).

There are several requirements for using nanoparticles as drugs, such as size (primary particle size, volume, and surface area), agglomeration state, distribution in two or three dimensions, chemical composition (element identification and distribution, crystal shape, and particle size distribution), and surface composition (charge on the surface). These requirements are crucial for nanoparticles used in biomedical applications. However, specifics of nanoparticle size in bulk materials and intended pharmaceutical items are critical to comprehending a drug’s pharmacodynamics and pharmacokinetic characteristics. There is a clear association between the engineering of nanoparticles, including manufacturing, and their impact on cell surface composition, morphology (size and shape), surface composition, and aggregation. Consequently, for employing green synthesized microalgal NPs for medical usage, more research is needed to ensure that these NPs can pass quality-control examinations. Additionally, essential quality characteristics, including solubility, stability, and solid-state qualities, should be considered when evaluating the suitability of green synthesized microalgal NPs for various pharmaceutical and medicinal applications (Taghizadeh et al., 2021).

## 9 Future perspectives

To date, only a few nanoparticles of gold and silver have been synthesized using microalgae through green synthesis. The green synthesis of zinc oxide, copper, selenium, titanium, and iron NPs of microalgae and its role in cancer treatment need to be explored. Additionally, numerous microalgae have a high potential for nanoparticle synthesis that remains unexplored. Therefore, microalgal NPs synthesis is a trending field that requires intense research for medicinal applications, especially for cancer treatment.

The microalgal NPs synthesis towards cancer treatment may benefit from further research on the contribution of synthetic biology and genetic engineering approaches. Synthetic biology intrusion enables precise genetic modifications through CRISPR/Cas9 technologies to manipulate the metabolic pathways, which help in the efficient conversion of metal ions into NPs with desired sizes and properties, which eventually help in the cost-effectiveness of the process, which suits the needs of the biomedical industry. The research domain also needs to explore the possibilities of utilizing genetically engineered microalgae for NPs synthesis for cancer treatment towards enhanced efficiencies with tailored NPs producing traits in a sustainable production manner (Zhang and Fussenegger, 2024).

## 10 Conclusion

The review discussed the role of microalgal NPs as therapeutic agents for treating cancer cells through a sustainable, green synthesis approach. Furthermore, the probable mechanism of microalgal NP’s synthesis, its characterization approaches, and technical challenges associated with the process have been discussed. Moreover, the extended application of green-synthesized microalgal NPs to cancer cells was addressed through the mechanism of action on cancer cells and cytotoxicity studies. Finally, the quality control aspects and prospects of green-synthesized microalgal NPs are summarized. This review provides a concise overview of the green synthesis of microalgal NPs for potential cancer treatment.
